# Effects of anesthetic adjunctive agents on postoperative cognitive dysfunction in elderly patients undergoing noncardiac surgery: A Bayesian network meta‐analysis

**DOI:** 10.1002/brb3.3149

**Published:** 2023-07-11

**Authors:** Lichi Xu, Shuxiang Xu, Yuelun Zhang, Yuguang Huang

**Affiliations:** ^1^ Department of Anesthesiology, Peking Union Medical College Hospital Chinese Academy of Medical Sciences & Peking Union Medical College Beijing P. R. China; ^2^ Department of Pain Management Shandong Provincial Hospital, Shandong University Jinan Shandong P. R. China; ^3^ Medical Research Center, Peking Union Medical College Hospital Chinese Academy of Medical Sciences & Peking Union Medical College Beijing P. R. China

**Keywords:** anesthetic adjunctive agents, Bayesian network meta‐analysis, elderly patients, postoperative cognitive dysfunction (POCD)

## Abstract

**Background:**

Elderly patients are prone to postoperative cognitive dysfunction (POCD). The comparison of the effects of anesthetic adjuvant drugs on POCD in elderly patients undergoing noncardiac surgery remains controversial.

**Methods:**

The final search took place on June 10, 2023. Randomized controlled trials including ketamine, ulinastatin, dexmedetomidine, parecoxib, and midazolam on the prevention and treatment of POCD in elderly undergoing noncardiac surgery were collected. A Bayesian network meta‐analysis was performed to quantitatively combine the evidence.

**Results:**

A total of 35 randomized trials were finally included in this systematic review, and the overall risk of bias is Allocation concealment. These anesthetic adjuvant drugs did not show significant differences in preventing POCD on postoperative days 1 and 7 compared with each other, but ulinastatin may be more effective in preventing POCD than dexmedetomidine [odds ratio (OR) = 0.28, 95% confidence interval (CI) = (0.10, 0.71)] and parecoxib [OR = 0.3, 95% CI = (0.10, 0.82 on postoperative day 3. The efficiency ranking results also find that ulinastatin and ketamine might provide better effects regarding POCD prevention.

**Conclusions:**

Ketamine and ulinastatin might have better effects in preventing POCD in elderly patients undergoing noncardiac surgery. Our meta‐analysis provided evidence for the use of ulinastatin and ketamine in the prevention of POCD in elderly patients undergoing noncardiac surgery.

## 1 INTRODUCTION

Postoperative cognitive dysfunction (POCD), defined as a decline of neurocognitive ability occurring after an operation, is a common postoperative complication in patients undergoing surgery (Brodier & Cibelli, 2021). The incidence of POCD is related to many factors such as surgical methods and the age of patients (Belrose & Noppens, 2019; Urits et al., 2019). The incidence of POCD in cardiac surgery is significantly higher than in noncardiac surgery (Czok et al., 2021). However, studies have shown that the incidence of POCD in older patients undergoing noncardiac surgery is significantly higher than in younger patients (Belrose & Noppens, 2019; Brodier & Cibelli, 2021; Urits et al., 2019). The incidence of POCD varies between 20 and 40% in patients over 60 years old (Lin et al., 2020). POCD is associated with longer hospital stay, impaired life function, which also increase postoperative mortality, but the drugs and methods for the prevention and treatment of POCD are still unclear (Brodier & Cibelli, 2021; Ruggiero et al., 2017). Therefore, it is important to develop new strategies to prevent POCD, especially in elderly patients.

In recent years, more and more randomized controlled clinical studies (RCTs) have suggested that there are various anesthetic drugs and anesthesia adjuvant drugs that have the effect of preventing and treating POCD (Duan et al., 2021; Glumac et al., 2017; Hovaguimian et al., 2018; Huang et al., 2020a; Li et al., 2020; Mansouri et al., 2019; Yu et al., 2022). The application of these drugs during the perioperative period could effectively prevent the occurrence of POCD (Duan et al., 2021; Glumac et al., 2017; Hovaguimian et al., 2018; Huang et al., 2020a; Li et al., 2020; Mansouri et al., 2019; Yu et al., 2022). Drugs with potential to prevent and treat POCD include dexmedetomidine, ketamine, ulinastatin, parecoxib, midazolam, lidocaine, and dexamethasone (Duan et al., 2021; Glumac et al., 2017; Hovaguimian et al., 2018; Huang et al., 2020a; Li et al., 2020; Mansouri et al., 2019; Yu et al., 2022). However, two recent meta‐analyses showed that lidocaine and dexamethasone did not have the effect of preventing POCD (Glumac et al., 2017; Huang et al., 2020b). Parecoxib is a selective Cox‐2 inhibitor, which belongs to nonsteroidal anti‐inflammatory drugs (NSAIDs) (Huang et al., 2020a). In NSAIDs, only selective Cox‐2 inhibitors are reported to prevent POCD by reducing the central nervous inflammatory response (Yagami et al., 2016). However, in our presearch process, it was found that only parecoxib reported by RCTs and meta‐analyses could be used to treat POCD occurring on postoperative days 3 and 7. Therefore, the efficacy of dexmedetomidine, ketamine, ulinastatin, and parecoxib in the prevention of POCD was supported by meta‐analyses (Duan et al., 2021; Hovaguimian et al., 2018; Huang et al., 2020a; Yu et al., 2022). However, there is no head‐to‐head comparison between these drugs above. The pharmacological effects and mechanisms of these drugs are different, and their efficacy for the prevention of POCD in the elderly remains controversial, which greatly affects the clinical applications of these drugs.

In this study, we sought to investigate the effect of dexmedetomidine, ketamine, ulinastatin, parecoxib, and midazolam on the incidence of POCD in elderly patients. Since the incidence of POCD was significantly different between cardiac surgery and noncardiac surgery, we only evaluated the prevention and treatment effects of these drugs on POCD in noncardiac surgery (Brodier & Cibelli, 2021; Czok et al., 2021). We found that neurocognitive tests are often performed on postoperative days 1, 3, and 7, according to the present RCTs (Duan et al., 2021; Huang et al., 2020a; Li et al., 2021a; Mansouri et al., 2019). In view of the different incidence rates of POCD at different time points after operation, we performed summary statistics and meta‐analyses on the occurrence of POCD on postoperative days 1, 3, and 7 (Duan et al., 2021; Huang et al., 2020a; Li et al., 2021a; Mansouri et al., 2019).

## 2 MATERIALS AND METHODS

The PRISMA 2020 (Preferred Reporting Project 2020 for Systematic Review and Meta‐Analysis) Statement was followed in the report of the study, and the Cochrane Handbook for Systematic Reviews of Interventions was followed in the design and conduct of the study (Cumpston et al., 2022; Page et al., 2021). Our study has been registered with PROSPERO (registration number: CRD42022362230).

### 2.1 Search strategy

Relevant studies were searched in Medline, Embase, Cochrane Library (Cochrane Central Registry of Controlled Trials), and CNKI databases using the following combination strategies with both MeSH terms and free‐text keywords including ((“perioperative cognitive dysfunction” [MeSH] OR “POCD” [MeSH] OR “ postoperative cognitive impairment” OR “postoperative neurocognitive dysfunction” [MeSH] OR “PND” [MeSH] OR “postoperative cognitive disorder”) AND (“Elderly patients” [MeSH] OR “Old Patients” [MeSH] OR “the Elderly” [MeSH]) AND (“An*esthetic Drugs” [MeSH] OR “An*esthetic Agents” [MeSH] OR “An*esthetic Effect” [MeSH] OR “Dexmedetomidine ” [MeSH] OR “Ketamine” [MeSH] OR “Ketalarum” OR “Ketaject” OR “Ulinastatin” [MeSH] OR “Parecoxib” [MeSH] OR “Dynastat” OR “Cox‐2 inhibitor” OR “Cox‐2 antagonist” OR “Midazolam” [MeSH] OR “ Dormicum”) AND (“Randomized Controlled Trial” [MeSH] OR “RCT” [MeSH] OR “Meta‐analysis” [MeSH])). The final search took place on June 10, 2023.

### 2.2 Study selection

Studies that met the following criteria were included:

1. Designed as a parallel group RCTs and RCTs included in the previous relevant meta‐analysis were also checked and included if they met our inclusion and exclusion criteria.
2. Included elderly patients (over 60 years old) undergoing noncardiac surgery who were randomly assigned to a treatment group.
3. Reported the incidence of POCD or the number of patients with POCD. The diagnostic criteria for POCD in our meta‐analysis were consistent with those of the studies included. Reviews and duplicate reports were excluded.

### 2.3 Data extraction and quality assessment

Database search, data extraction, and quality assessment were performed by two independent authors. In case of disagreement, the two authors will negotiate with the corresponding author. We extracted the study information (including first author, year of publication, and the country of the study), study design, patient information, type of surgery, and diagnostic strategy for POCD (postoperative neurocognitive function test method). The Cochrane Risk of Bias Tool (version 1) was used for quality assessment based on the following aspects: (1) Random sequence generation; (2) allocation concealment; (3) blinding of participants and personnel; (4) blinding of outcome assessment; (5) incomplete outcome data addressed; (6) selective results reporting (Cumpston et al., 2022).

### 2.4 Network meta‐analysis

We used Stata software to make the figure of the network evidence. The nodes of the figure represent the drugs compared, with size according to sample size, connected by edges whose thickness is proportional to the number of trials. The random‐effects model was used for statistical analysis in our study (Higgins et al., 2003). We performed Bayesian network meta‐analysis by using the statistical package “gemtc” in R software. Markov chain Monte Carlo method is used to create samples with the chain number set to 4, and optimization iteration number set to 20,000. An odds ratio (OR) with a 95% confidence interval (95% CI) was used to determine the incidence of POCD. The Brooks–Gelman–Rubin convergence diagnosis was used to evaluate convergence (Brooks et al., 1998). The *X* ^2^ test was used to test heterogeneity and *I* ^2^ was used for quantitative analysis. *I* ^2^ less than 50% indicate that there is homogeneity between studies, while *I* ^2^ ≥ 50% indicates that there exists heterogeneity among studies. One of the main assumptions of network meta‐analysis is the inconsistency test between direct and indirect comparison. If a loop connecting the three arms is found, the inconsistency test is performed using the node splitting method (Naci & Fleurence, 2011). A *p* value greater than .05 is considered to be consistent. We ranked the efficacy of the drugs above according to the results of the network meta‐analysis and drew the efficacy ranking figures. Stata software was used to draw funnel plots to compare the publication bias.

## 3 RESULTS

### 3.1 Literature search results

**FIGURE 1:**
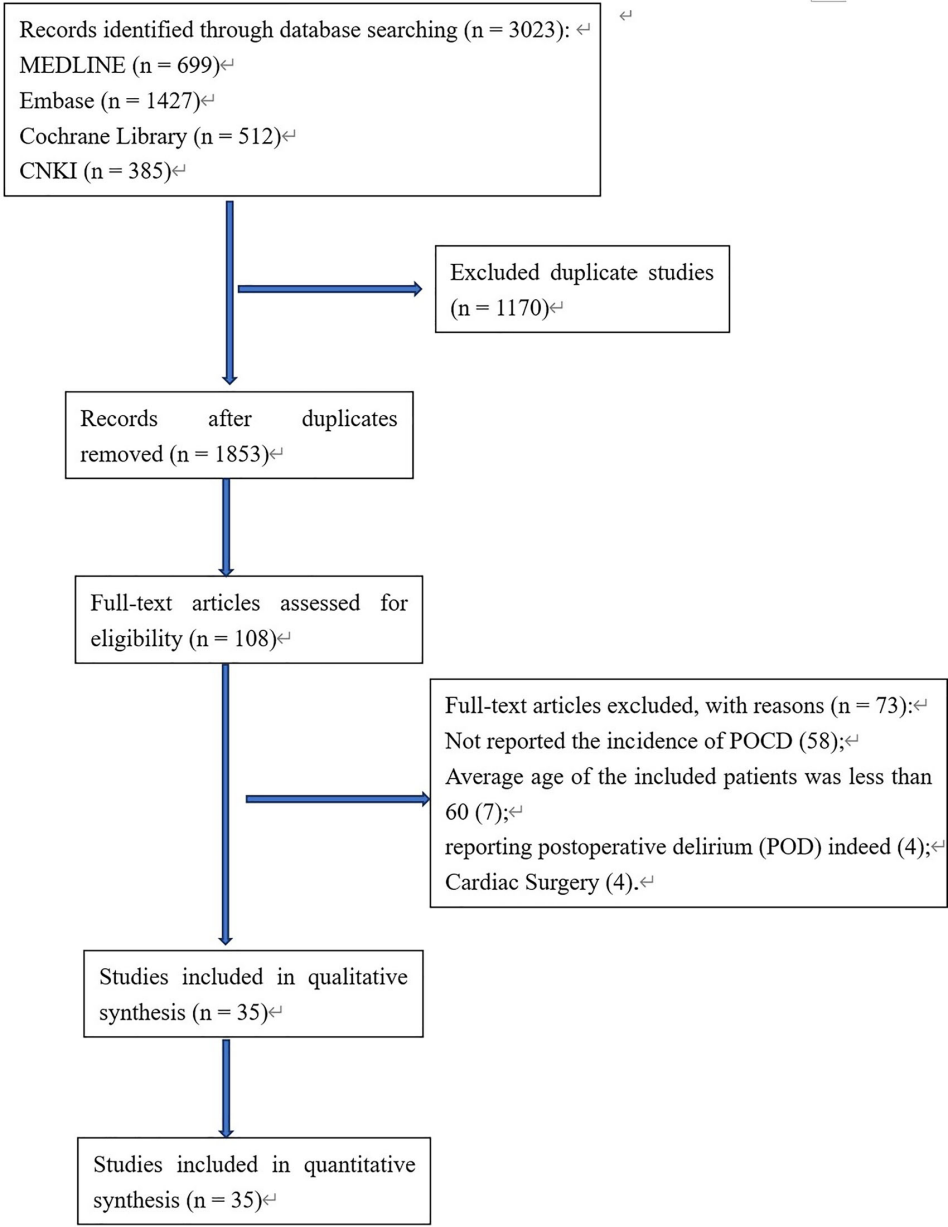
Flow chart of literature search.

**FIGURE 2:**
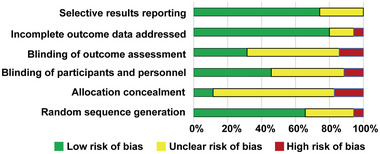
Risk of bias graph. Review authors’ judgments about each risk of bias item presented as percentages across all included studies.

**FIGURE 3:**
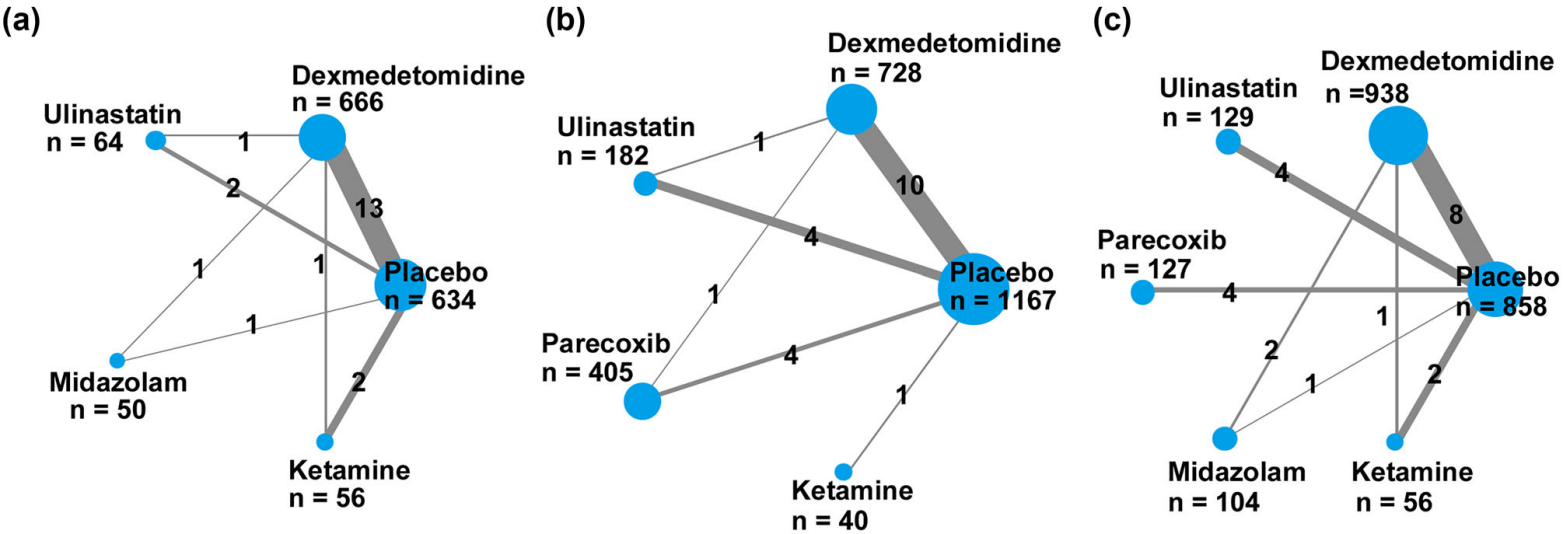
Network of randomized controlled trials comparing different adjuvant drugs for preventing POCD in elderly patients undergoing noncardiac surgery on postoperative day 1 **(a)**, postoperative day 3 **(b),** and postoperative day 7 **(c)**. The thickness of the connecting lines represents the number of trials between each comparator, and the size of each node corresponds to the number of subjects (sample size).

We searched 699 articles in Medline, 1427 articles in Embase, 512 articles in the Cochrane Library, and 385 articles in CNKI. A total of 1853 studies were identified after excluding the duplicated studies. After reviewing their titles and abstracts, 1745 studies were excluded. The remaining 108 studies were evaluated in detail by reading the full text. A total of 58 studies were excluded due to the absence of the number of patients with POCD or the incidence of POCD; 7 studies were excluded due to unsatisfied ages of patients; 4 studies were excluded because of reporting postoperative delirium (POD) indeed; 4 studies were excluded because of containing cardiac surgery. The flow chart for the retrieval of the literature is shown in Figure 1. Finally, 35 studies were used for the final synthesis of the data, involving 3431 subjects. A total of 21 studies involve dexmedetomidine; 7 studies involve parecoxib; 7 studies involve ulinastatin; 3 studies involve ketamine; and 2 studies involve midazolam. The clinical characteristics of the studies above are shown in Supporting Information Table S1. Figure 2 shows the risk of bias of the studies included in this meta‐analysis. The results of the evaluation of the risk of bias of the studies are shown in Supporting Information Table S2. Since there are three time points for measuring POCD in the studies, we used each time point as an outcome to screen relevant studies in our Bayesian network meta‐analysis. Figure 3 shows the network evidence of three time points for POCD.

### 3.2 Network meta‐analysis

Table 1 shows the result of mutual comparison in our network meta‐analysis. On postoperative day 3, ketamine [OR = 0.16, 95% CI = (0.02, 0.83)], ulinastatin [OR = 0.11, 95% CI = (0.04, 0.25)], dexmedetomidine [OR = 0.40, 95% CI = (0.26, 0.56)], and parecoxib [OR = 0.36, 95% CI = (0.20, 0.57)] have the effects of preventing POCD. Ulinastatin may be more effective in preventing POCD than dexmedetomidine [OR = 0.28, 95% CI = (0.1, 0.71)] and parecoxib [OR = 0.3, 95% CI = (0.10, 0.82)] on postoperative day 3 but not than ketamine [OR = 0.69, 95% CI = (0.10, 7.15)]. On postoperative day 1, ketamine [OR = 0.13, 95% CI = (0.01, 0.64)], ulinastatin [OR = 0.19, 95% CI = (0.06, 0.53)], and dexmedetomidine [OR = 0.33, 95% CI = (0.23, 0.47)] have the effects of preventing POCD, while midazolam [OR = 0.45, 95% CI = (0.13, 1.35)] does not have the effect of preventing POCD compared with placebo. However, these anesthetic adjuvant drugs above did not show significant differences in preventing POCD on postoperative day 1. On postoperative day 7, ulinastatin [OR = 0.21, 95% CI = (0.09, 0.45)] and dexmedetomidine [OR = 0.44, 95% CI = (0.28, 0.67)] have the effects of preventing POCD while ketamine [OR = 0.13, 95% CI = (0, 1.14)], parecoxib [OR = 0.57, 95% CI = (0.30, 1.12)], and midazolam [OR = 0.52, 95% CI = (0.22, 1.20)] do not have the effect of preventing POCD compared with placebo. Ulinastatin may be more effective in preventing POCD than parecoxib [OR = 0.37, 95% CI = (0.12, 0.96)] on postoperative day 7 while other drugs did not show a significant difference. Forest plots of OR and 95% CI are shown in Supporting Information Figures S1 to 3, and we could see the mutual comparison of the effects of the drugs on postoperative days 1, 3, and 7 directly.

**TABLE 1:** Network meta‐analysis comparisons.

On postoperative day 1 [OR (95% CI)]					
	Placebo	Dexmedetomidine	Ulinastatin	Midazolam	Ketamine
Placebo	1	**.33 (.23,.47)**	**.19 (.06, .53)**	.45 (.13, 1.35)	**.13 (.01, .64)**
Dexmedetomidine	**3.03 (2.12, 4.35)**	1	.59 (.18, 1.68)	1.37 (.4, 4.36)	.39 (.04, 1.98)
Ulinastatin	**5.17 (1.88, 16.18)**	1.7 (.59, 5.41)	1	2.3 (.49, 11.54)	.64 (.06, 5.15)
Midazolam	2.2 (.74, 7.43)	.73 (.23, 2.51)	.43 (.09, 2.04)	1	.28 (.02, 2.01)
Ketamine	**7.83 (1.57, 76.56)**	2.58 (.5, 24.24)	1.56 (.19, 17.08)	3.61 (.5, 44.29)	1

On postoperative day 3 [OR (95% CI)]					
	Placebo	Dexmedetomidine	Ulinastatin	Parecoxib	Ketamine
Placebo	1	**.4 (.26, .56)**	**.11 (.04, .25)**	**.36 (.2, .57)**	**.16 (.02, .83)**
Dexmedetomidine	**2.5 (1.77, 3.92)**	1	**.28 (.1, .71)**	.9 (.49, 1.67)	.4 (.04, 2.18)
Ulinastatin	**9.07 (3.98, 24.82)**	**3.61 (1.42, 10.14)**	1	**3.29 (1.22, 9.72)**	1.45 (.14, 9.91)
Parecoxib	**2.77 (1.76, 4.9)**	1.11 (.6, 2.04)	**.3 (0.1, .82)**	1	.45 (.05, 2.57)
Ketamine	**6.34 (1.21, 58.54)**	2.48 (0.46, 23.02)	.69 (0.1, 7.15)	2.24 (.39, 22.14)	1

On postoperative day 7 [OR (95% CI)]						
	Placebo	Dexmedetomidine	Ulinastatin	Parecoxib	Midazolam	Ketamine
Placebo	1	**0.44 (.28, .67)**	**.21 (.09, .45)**	.57 (.3, 1.12)	.52 (.22, 1.2)	.13 (0, 1.14)
Dexmedetomidine	**2.3 (1.5, 3.58)**	1	.49 (.18, 1.13)	1.32 (.61, 3.03)	1.2 (.52, 2.58)	.3 (.01, 2.57)
Ulinastatin	**4.76 (2.22, 11.24)**	2.06 (.88, 5.46)	1	**2.70 (1.01, 8.12)**	2.5 (.76, 8.24)	.62 (.02, 6.76)
Parecoxib	1.74 (.9, 3.34)	.76 (.33, 1.63)	**.37 (.12, .96)**	1	.91 (.3, 2.57)	.22 (.01, 2.23)
Midazolam	1.91 (.83, 4.62)	.83 (.39, 1.91)	.4 (.12, 1.31)	1.1 (.39, 3.3)	1	.25 (.01, 2.43)
Ketamine	7.48 (.88, 242.7)	3.34 (.39, 103.5)	1.62 (.15, 56.9)	4.53 (.45, 164.2)	4.02 (.41, 137.3)	1

**FIGURE 4:**
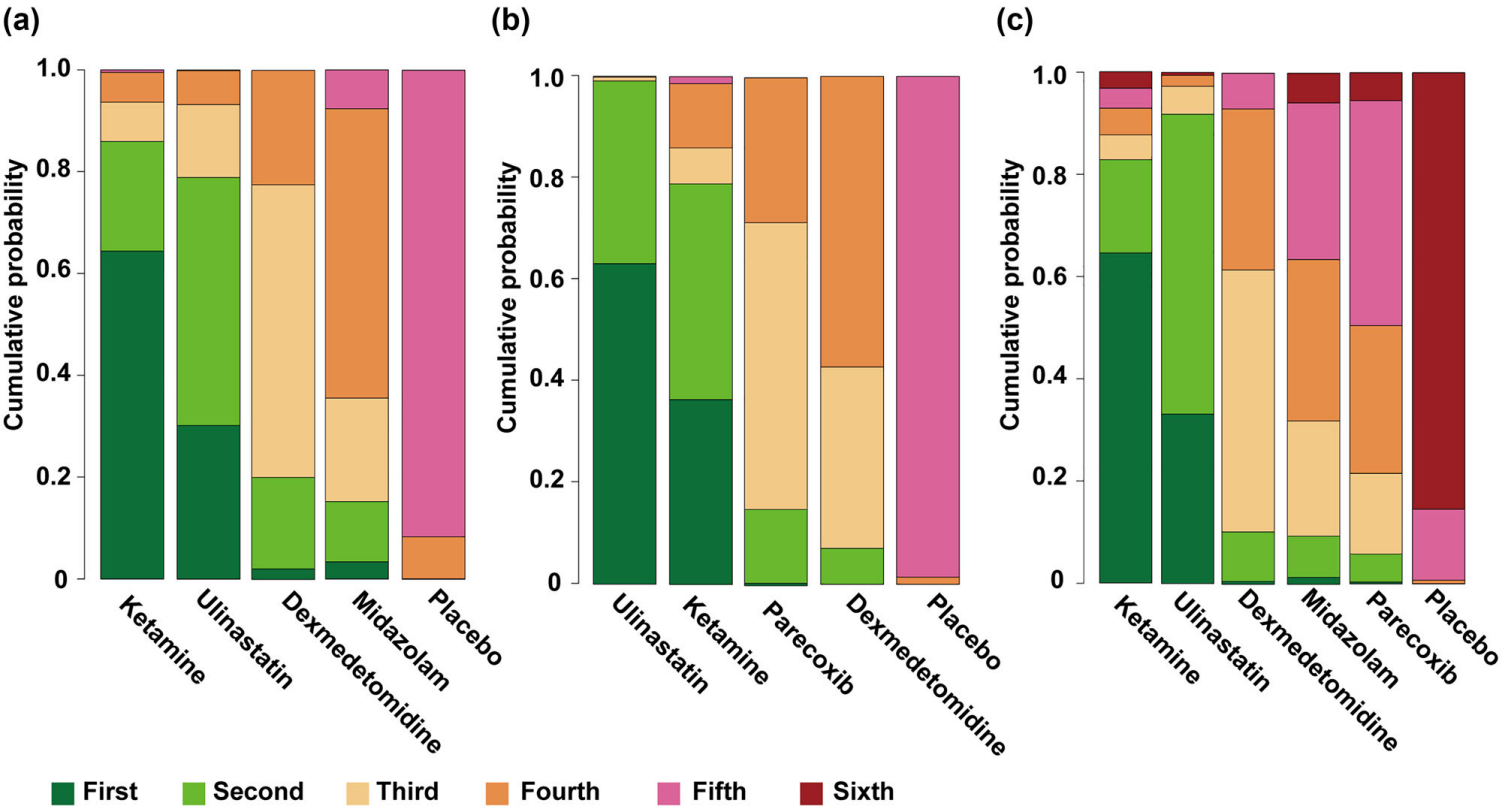
A rankogram of the probabilities of each drug in preventing POCD on postoperative day 1 **(a)**, postoperative day 3 **(b),** and postoperative day 7 **(c)**.

The efficacy probability ranking plot was drawn according to the efficacy ranking probability of each drug in preventing POCD (Figure 4). The order of efficacy of the drugs for the prevention of POCD on postoperative day 1 was ketamine, ulinastatin, dexmedetomidine, parecoxib, midazolam, and placebo (Figure 4a), and the order of efficacy was ulinastatin, ketamine, parecoxib, dexmedetomidine, and placebo on postoperative day 3 (Figure 4b). On postoperative day 7, the order of efficacy was ketamine, ulinastatin, dexmedetomidine, midazolam, parecoxib, and placebo (Figure 4c). The ranking probability values for each drug are shown in Supporting Information Table S3.

### 3.3 Inconsistency test and publication bias evaluation

**FIGURE 5:**
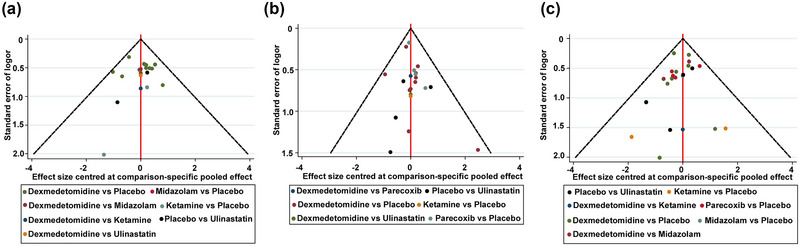
Funnel plots for the publication bias within the meta‐analysis of effect of different adjuvant drugs for preventing POCD in elderly patients undergoing noncardiac surgery on postoperative day 1 **(a)**, postoperative day 3 **(b),** and postoperative day 7 **(c)**.

We summarized the heterogeneity test and the OR values of direct comparison, indirect comparisons, and network comparison in Supporting Information Table S4. The heterogeneities are low among studies in most of the comparisons. Most of the results of direct and indirect comparisons were in good consistency (*p* > .05), indicating that the results of this mesh meta‐analysis were reliable (Supporting Information Table S4). However, there exists inconsistency between dexmedetomidine and ketamine in the efficacy on the prevention of POCD on postoperative day 7 (*p* < .001). Comparison of efficacy between dexmedetomidine and ketamine should be interpreted with caution. Inverted funnel plots were made according to publication bias (Figure 5). The data points were evenly distributed on both sides of the inverted funnel plot, and no obvious funnel plot asymmetry was observed.

## 4 DISCUSSION

In this study, we used a Bayesian network model to compare the effects of dexmedetomidine, ketamine, ulinastatin, parecoxib, and midazolam on the prevention of POCD on postoperative days 1, 3, and 7 in elderly patients undergoing noncardiac surgery. The results showed that the effects of ketamine, ulinastatin, dexmedetomidine, and parecoxib were better than placebo. Therefore, these four drugs could reduce the incidence of POCD in elderly patients undergoing noncardiac surgery which could be used in the prevention and treatment of POCD during the perioperative period. Our meta‐analysis showed that midazolam was no better than placebo in preventing POCD, and more clinical studies are needed in the future to confirm the efficacy of midazolam in preventing POCD in elderly patients undergoing noncardiac surgery.

POCD, defined as a decline of neurocognitive ability occurring after operation, is a common postoperative complication in patients undergoing surgery (Brodier & Cibelli, 2021). POCD and POD both belong to perioperative neurocognitive dysfunction (PND) (Safavynia & Goldstein, 2018). The characteristic manifestations of POCD are subtle and multifaceted, involving multiple cognitive domains such as attention, memory, and executive function (Krenk et al., 2010; Rasmussen, 2006). The most common problems of POCD are memory impairment and intellectual impairment (Rundshagen, 2014). The diagnosis of POCD requires preoperative and postoperative psychological testing (Zhang et al., 2019). For example, testing Mini‐mental State Examination (MMSE) before and after surgery, and POCD can be diagnosed with significant cognitive decline after surgery compared to the preoperative period (Zhang et al., 2019). POD is a common neuropsychiatric postoperative complication, mainly in elderly patients (Kukreja et al., 2015). POD is often identified by a series of neuropsychiatric tests, such as CAM and ICU‐CAM, which also indicates that POD often occurs in patients admitted to the ICU after surgery (Deiner et al., 2017; Mu et al., 2017). POD usually occurs 1–3 days after surgery, and the onset is sudden (Evered et al., 2018). However, studies we included found that POCD also occurred on postoperative days 1, 3, and 7, and can be diagnosed by neurocognitive tests (Chen et al., 2013a; Li et al., 2021b). The studies included are focused on POCD that occurred on postoperative days 1, 3, and 7 rather than POD. POD is also harmful to patients, and more RCTs are needed in the future to study drug therapy to prevent POD.

As shown in Table 1, ulinastatin may be more effective in preventing POCD than dexmedetomidine and parecoxib on postoperative day 3, and the order of efficacy of the four drugs on postoperative day 3 was ulinastatin, ketamine and parecoxib, dexmedetomidine, and placebo. These results indicated that ulinastatin had significant effects on preventing POCD in elderly patients undergoing noncardiac surgery. According to the efficiency ranking results on postoperative days 1 and 7, ketamine might provide better effect in preventing POCD in elderly patients undergoing noncardiac surgery. Therefore, the preventive effect of ketamine and ulinastatin on POCD in elderly patients undergoing noncardiac surgery deserves clinical attention.

Ketamine is a noncompetitive antagonist of the NMDA receptor that exerts anesthetic and analgesic effects by antagonizing NMDA receptors (Lee et al., 2015). In clinical studies, ketamine has been found to be effective in refractory status epilepticus, reducing the occurrence of disseminated depolarization in various brain disorders, and suppressing systemic inflammatory responses (Beilin et al., 2007; Hertle et al., 2012; Hovaguimian et al., 2018; Zeiler et al., 2014). Several preclinical studies have shown that ketamine acts as a neuroprotective agent at the extracellular and intracellular levels (Bell, 2017; Li & Vlisides, 2016; Urban et al., 2008). Ketamine appears to reduce the release of proinflammatory cytokines, prevent microthrombosis, promote neuronal growth, and reduce calcium‐mediated cell death and excitotoxicity (Bell, 2017; Li & Vlisides, 2016; Urban et al., 2008). Evidence of the neuroprotective effect of ketamine has also been described in pediatric surgery studies, where ketamine reduces the risk of agitation in pediatric patients in a variety of surgeries (Bilgen et al., 2014; Chen et al., 2013b; Eghbal et al., 2013; Ozturk et al., 2016).

Ulinastatin is a protease inhibitor that reduces the inflammatory response through antioxidant responses, antiproteolysis, and inhibition of the release of inflammatory mediators (Lv et al., 2020). Ulinastatin reduces the postoperative neuroinflammatory response by inhibiting the release of neuroinflammatory factors caused by surgical trauma, which reduces the incidence of POCD (Lv et al., 2016). Clinical studies and meta‐analysis suggest that ulinastatin may attenuate POCD by reducing the release of TNF‐α and IL‐6 (Duan et al., 2021; Hirsch et al., 2016; Li et al., 2018; Lili et al., 2013). Ulinastatin reduces proinflammatory cytokine levels, such as TNF‐α, CRP, and IL‐6, by activating the PI3K/Akt/Nrf2 pathway (Li et al., 2018). It also promotes the release of anti‐inflammatory cytokine IL‐10 by inhibiting the JNK/NF‐κB pathway (Li et al., 2018). Ulinastatin has been shown to upregulate the anti‐inflammatory factor IL‐10, which is associated with the attenuation of POCD (Lili et al., 2013; Wang et al., 2017). Studies have also suggested that ulinastatin attenuates the increase of S100β and the incidence of POCD, whose mechanism may be the reduction of serum levels of IL‐6 and CRP and the increase of IL‐10 levels (Lili et al., 2013).

Li et al. (2020) conducted a network meta‐analysis to compare the effects of dexmedetomidine, ketamine, lidocaine, and dexamethasone on the prevention of POCD in patients undergoing surgery under general anesthesia. They found that dexmedetomidine may decrease POCD when compared with placebo [OR = 3.1, 95% CI = (1.8, 6.4)] for the noncardiac surgery which is consistent with our study. However, they found that ketamine, lidocaine, and dexamethasone did not show a statistically significant difference. According to our results, ulinastatin may be more effective in preventing POCD than dexmedetomidine and parecoxib on postoperative day 3, and ketamine may have the effects in preventing POCD compared with placebo on postoperative days 1 and 3. We believe that their results are different from ours for the following reasons: (1) In (Li et al.’s, 2021a) study, the detection time points of POCD were not analyzed separately, and the incidence of POCD was different at different time points after surgery. (2) Our network meta‐analysis contains six studies concerning dexmedetomidine in 2020 and 2021. (3) The drugs they focused on are dexmedetomidine, lidocaine, ketamine, and dexamethasone, while the drugs in our study are ketamine, ulinastatin, dexmedetomidine, parecoxib, and midazolam.

Although our network meta‐analysis included patients over 60 years of age who underwent noncardiac surgery and analyzed the occurrence of POCD on postoperative days 1, 3, and 7 separately, the results of our network meta‐analysis should be applied in clinical practice with caution. First, we did not analyze the application dose and application time of each drug separately. Due to the different targets of these drugs, their titer intensity is difficult to unified, and further stratified analysis will reduce the sample size and further reduce the reliability of the results (Cumpston et al., 2022; Page et al., 2021). Our study is the first Bayesian network meta‐analysis to investigate the preventive effect of these drugs on POCD. In the future, more RCT studies are needed to be done to confirm the preventive effect of these drugs on POCD in elderly patients. Second, due to the difference in the incidence of POCD between cardiac surgery and noncardiac surgery, we excluded relevant studies of POCD after cardiac surgery (Gao et al., 2020; Ge et al., 2015; Hudetz et al., 2009; Li et al., 2021a). Studies on the application of these drugs in elderly patients undergoing cardiac surgery are too few to perform a Bayesian network meta‐analysis. With the increase in elderly patients undergoing cardiac surgery, the effect of these drugs on the prevention of POCD in elderly patients undergoing cardiac surgery needs more research attention. Third, as for the limitations of the included studies or the review process itself, our included studies have heterogeneities such as different cognitive evaluation tools (Lili et al., 2013; Xu et al., 2017; Zhang et al., 2019; Zhu et al., 2016). Different neurocognitive evaluation tools may have different cognitive evaluation results (Li et al., 2021a; Yu et al., 2022). Most of our included studies used MMSE to evaluate the appearance of POCD which is a widely used neurocognitive evaluation method in POCD diagnosis (Li et al., 2021a). Although the funnel plot did not suggest significant publication bias, we should still be careful about the potential for publication bias in POCD studies. Our research searched the English databases, including Medline, Embase, Cochrane Library, and the Chinese database CNKI, covering the mainstream international medical research databases. Other language databases were not searched due to language barriers. Fourth, our results showed that dexmedetomidine and ketamine were significantly inconsistent in the prevention of POCD on postoperative day 7. Therefore, the preventive effects of dexmedetomidine and ketamine on POCD in elderly patients undergoing noncardiac surgery, especially on postoperative day 7, should be evaluated by more RCT studies. Nevertheless, our study provides a new idea for the drug prevention of POCD in elderly patients undergoing noncardiac surgery. It should be noted that the combination of the above drugs may have a better effect in preventing POCD, such as dexmedetomidine, combined with ulinastatin and dexmedetomidine combined with parecoxib (Du et al., 2019; Lu et al., 2017; Zhou et al., 2019). Several RCTS showed that the combination of the two drugs had a better effect on preventing POCD (Du et al., 2019; Lu et al., 2017; Zhou et al., 2019). The efficacy evaluation of the combined application of anesthesia adjuvant drugs in the prevention of POCD in elderly patients deserves further study in the future.

## 5 CONCLUSIONS

In conclusion, we compared the preventive effects of ketamine, ulinastatin, dexmedetomidine, parecoxib, and midazolam on POCD in elderly patients undergoing noncardiac surgery. Ketamine and ulinastatin might have better effects in preventing POCD in the elderly undergoing noncardiac surgery. The preventive effects of ketamine and ulinastatin on POCD in elderly patients deserve attention, and more large‐scale RCT studies and meta‐analyses are needed to demonstrate their effects and safety. The studies of ketamine and ulinastatin in the future are important which will validate our results and explore optimal dosing and administration protocols of these two drugs to provide more effective and safe perioperative medication regimen for elderly patients.

## CONFLICT OF INTEREST STATEMENT

The authors declare that the research was conducted in the absence of any commercial or financial relationships that could be construed as a potential conflict of interest.

### PEER REVIEW

The peer review history for this article is available at https://publons.com/publon/10.1002/brb3.3149

## CLINICAL TRIAL REGISTRATION

Our study has been registered with PROSPERO (registration number: CRD42022362230).

## Supporting information


**Supplementary Table 1**. Characteristics of the included RCTs.
**Supplementary Table 2**. Details of quality evaluation of the included RCTs via the Cochrane's Risk of Bias Tool.
**Supplementary Table 3**. Rank probabilities of each drug in preventing POCD.
**Supplementary Table 3A**. Rank probabilities of each drug in preventing POCD on postoperative Day 1.
**Supplementary Table 3B**. Rank probabilities of each drug in preventing POCD on postoperative Day 3.
**Supplementary Table 3C**. Rank probabilities of each drug in preventing POCD on postoperative Day 7.
**Supplementary Table 4**. Summary odds ratios and heterogeneity for each comparison.
**Supplementary Table 4A**. Summary odds ratios and heterogeneity for each comparison on postoperative Day 1.
**Supplementary Table 4B**. Summary odds ratios and heterogeneity for each comparison on postoperative Day 3.
**Supplementary Table 4C**. Summary odds ratios and heterogeneity for each comparison on postoperative Day 7.
**Supplementary Figure 1**. Forest plots of odds ratios (95% confidence interval) produced by network meta‐analysis postoperative Day 1.
**Supplementary Figure 2**. Forest plots of odds ratios (95% confidence interval) produced by network meta‐analysis postoperative Day 3.
**Supplementary Figure 3**. Forest plots of odds ratios (95% confidence interval) produced by network meta‐analysis postoperative Day 7.

## Data Availability

The original contributions presented in the study are included in the article/Supplementary Material, further inquiries can be directed to the corresponding author.
