# Supplementary material for: Effects of anesthetic adjunctive agents on postoperative cognitive dysfunction in elderly patients undergoing noncardiac surgery: A Bayesian network meta‐analysis
Source: Brain Behav. 2023 Jul 11;13(8):e3149. doi: 10.1002/brb3.3149 (PMC10454282; doi:10.1002/brb3.3149)
Supplement: Supplementary file 1 — Supplementary Table 1. Characteristics of the included RCTs. Supplementary Table 2. Details of quality evaluation of the included RCTs via the Cochrane's Risk of Bias Tool. Supplementary Table 3. Rank probabilities of each drug in preventing POCD. Supplementary Table 3A. Rank probabilities of each drug in preventing POCD on postoperative Day 1. Supplementary Table 3B. Rank probabilities of each drug in preventing POCD on postoperative Day 3. Supplementary Table 3C. Rank probabilities of each drug in preventing POCD on postoperative Day 7. Supplementary Table 4. Summary odds ratios and heterogeneity for each comparison. Supplementary Table 4A. Summary odds ratios and heterogeneity for each comparison on postoperative Day 1. Supplementary Table 4B. Summary odds ratios and heterogeneity for each comparison on postoperative Day 3. Supplementary Table 4C. Summary odds ratios and heterogeneity for each comparison on postoperative Day 7. Supplementary Figure 1. Forest plots of odds ratios (95% confidence interval) produced by network meta‐analysis postoperative Day 1. Supplementary Figure 2. Forest plots of odds ratios (95% confidence interval) produced by network meta‐analysis postoperative Day 3. Supplementary Figure 3. Forest plots of odds ratios (95% confidence interval) produced by network meta‐analysis postoperative Day 7. [file BRB3-13-e3149-s001.pdf]

Supplementary Material

1 Supplementary Tables

Supplementary Table 1. Characteristics of the included RCTs.

Author	Country	Treatment 1		Events of POCD			Treatment 2		Events of POCD			Treatment 3		Events of POCD			Surgical type	Cognitive testing methods
		Drug	Age (Mean)	1 day	3 days	7 days	Drug	Age (Mean)	1 day	3 days	7 days	Drug	Age (Mean)	1 day	3 days	7 days		
Chen 2013	China	Placebo	67.9	27/63	NA	NA	Dexmedetomidine	66.2	9/59	NA	NA						Laparoscopic cholecystectomy	MMSE
Guan 2015	China	Placebo	68.2	8/30	9/30	NA	Dexmedetomidine	65.9	9/30	10/30	NA						Laparoscopic surgery	MMSE
Kong 2018	China	Placebo	68.9	17/60	NA	NA	Dexmedetomidine	67.8	6/60	NA	NA						Cancer surgery	MMSE
Xu 2017	China	Placebo	72.1	2/48	2/48	NA	Dexmedetomidine	71.9	0/48	1/48	NA						Ovarian cystectomy	MoCA
Zhang 2019	China	Placebo	74.09	13/60	8/60	NA	Dexmedetomidine	73.76	7/80	0/80	NA						Radical colorectal cancer surgery	MMSE
Peng 2012	China	Placebo	67.2	NA	11/40	NA	Dexmedetomidine	70.6	NA	5/40	NA						Prostate resection	MMSE
Li 2015	China	Placebo	70	21/50	NA	NA	Dexmedetomidine	69	10/50	NA	NA						Laparoscopic cholecystectomy	MMSE
Mohamed 2014	Africa	Placebo	67.8	20/25	NA	NA	Dexmedetomidine	63.9	4/25	NA	NA						Abdominal surgery	MoCA

Continuation table of **Supplementary Table 1.**

Author	Country	Treatment 1		Events of POCD			Treatment 2		Events of POCD			Treatment 3		Events of POCD			Surgical type	Cognitive testing methods
		Drug	Age (Mean)	1 day	3 days	7 days	Drug	Age (Mean)	1 day	3 days	7 days	Drug	Age (Mean)	1 day	3 days	7 days		
Wang 2020	China	Placebo	68.26	20/50	NA	NA	Dexmede-tomidine	68.37	12/60	NA	NA						Radical gastrectomy	MMSE
Shi 2020 1	China	Placebo	68.7	19/53	NA	NA	Dexmede-tomidine	68.71	7/53	NA	NA						Thoracoscopic lobectomy	MMSE
Shi 2020 2	China	Placebo	68.7	NA	12/40	NA	Dexmede-tomidine	68.71	NA	4/40	NA						Thoracoscopic lobectomy	MMSE
Zhao 2020	China	Placebo	69.2	NA	NA	30/101	Dexmede-tomidine	69.4	NA	NA	40/315						Non-cardiac surgery	MMSE
Li 2021	China	Placebo	73.4	13/30	12/30	12/30	Dexmede-tomidine	71.2	19/90	17/90	17/90						Spine surgery	MMSE
Liu 2020	China	Placebo	68.6	NA	NA	7/24	Dexmede-tomidine	69.6	NA	NA	5/24						Colorectal cancer radical resection	MMSE
Cheng 2019	China	Placebo	70	NA	65/266	49/266	Dexmede-tomidine	71	NA	40/269	31/269						Gastrointestinal laparotomy	MoCA
Zhang 2014	China	Placebo	71.5	NA	7/20	7/20	Dexmede-tomidine	71.6	NA	8/60	6/60						Laparoscopic surgery	MMSE
Shan 2015	China	Placebo	75	9/27	7/27	NA	Ulinastat-in	78	1/21	0/21	NA						Hip fracture surgery	MMSE
Ge 2011	China	Placebo	75	NA	22/80	NA	Ulinastat-in	72.8	NA	3/80	NA						Hip joint replacement	MMSE

Continuation table of **Supplementary Table 1.**

Author	Country	Treatment 1		Events of POCD			Treatment 2		Events of POCD			Treatment 3		Events of POCD			Surgical type	Cognitive testing methods
		Drug	Age (Mean)	1 day	3 days	7 days	Drug	Age (Mean)	1 day	3 days	7 days	Drug	Age (Mean)	1 day	3 days	7 days		
Kang 2010	China	Placebo	72.8	NA	10/40	NA	Ulinastatin	75	NA	1/40	NA						Hip joint replacement	MMSE
Wang 2017	China	Placebo	67	NA	NA	17/37	Ulinastatin	66	NA	NA	9/38						One lung ventilation surgery	MMSE
Zhang 2018	China	Placebo	71.3	NA	NA	13/30	Ulinastatin	72.8	NA	NA	5/30						Spine surgery	MoCA
Pan 2016	China	Placebo	73.9	13/41	9/41	NA	Ulinastatin	72.4	5/41	3/41	NA	Dexmedetomidine	71.9	7/41	4/41	NA	Laparoscopic colorectal cancer surgery	MMSE
Xu 2013	China	Placebo	74.1	NA	NA	11/40	Ulinastatin	75.6	NA	NA	1/40						Abdominal surgery	A battery of nine neuropsychological tests
Liu 2014	China	Placebo	70.1	NA	10/30	NA	Parecoxib	68.4	NA	3/30	NA						Hip replacement operation	MMSE
Tian 2015	China	Placebo	71.8	NA	19/35	NA	Parecoxib	72.5	NA	10/35	NA						Total hip replacement or femoral head resurfacing	MMSE

Continuation table of **Supplementary Table 1.**

Author	Count-ry	Treatment 1		Events of POCD			Treatment 2		Events of POCD			Treatment 3		Events of POCD			Surgical type	Cognitive testing methods
		Drug	Age (Mean)	1 day	3 days	7 days	Drug	Age (Mean)	1 day	3 days	7 days	Drug	Age (Mean)	1 day	3 days	7 days		
Zhao 2012	China	Placebo	70.5	NA	NA	6/20	Parecoxib	71.1	NA	NA	8/22						Selective knee arthroplasty	MMSE
Jin 2013	China	Placebo	72.5	NA	NA	7/30	Parecoxib	73.1	NA	NA	6/30						Orthopedic operation	MMSE
Zhu 2016	China	Placebo	74.3	NA	NA	21/45	Parecoxib	75.1	NA	NA	10/47						Selective knee arthroplasty	An test battery including 7 tests with 9 subscales
Zhang 2014	China	Placebo	72.3	NA	NA	10/30	Parecoxib	72.3	NA	NA	9/30						Selective knee arthroplasty	MMSE
Wu 2016	China	Placebo	64	NA	16/30	NA	Parecoxib	63	NA	8/30	NA	Dexmede-tomidine	66	NA	9/30	NA	Femoral head and total hip or total knee replacement	MoCA
Mans-ouri 2019	Iran	Placebo	64.02	12/50	NA	10/50	Midazol-am	63.6	7/50	NA	4/50	Dexmede-tomidine	66.5	6/50	NA	6/50	Cataract surgery	MMSE

Continuation table of **Supplementary Table 1.**

Author	Count-ry	Treatment 1		Events of POCD			Treatment 2		Events of POCD			Treatment 3		Events of POCD			Surgical type	Cognitive testing methods
		Drug	Age (Mean)	1 day	3 days	7 days	Drug	Age (Mean)	1 day	3 days	7 days	Drug	Age (Mean)	1 day	3 days	7 days		
Li 2019	China	Dexmedet-omidine	69.3	NA	NA	22/55	Midazol-am	66.9	NA	NA	28/54						Hip or knee arthroplasty	MoCA
Lee 2015	Korea	Placebo	68.38	0/25	NA	0/25	Ketamine	68.32	0/26	NA	1/26						Orthopedic surgery	MMSE
She 2017	China	Placebo	68.4	NA	9/40	NA	Ketamine	60.7	NA	2/40	NA						Laparoscopic cholecystecto-my	MMSE
Zhang 2013	China	Placebo	69.2	8/30	NA	4/30	Ketamine	65.8	2/30	NA	0/30	Dexmede-tomidine	69.1	6/30	NA	3/30	Orthopedic operation	MMSE

Supplementary Table 2. Details of quality evaluation of the included RCTs via the Cochrane's Risk of Bias Tool.

	Random sequence generation	Allocation concealment	Blinding of participants and personnel	Blinding of outcome assessment	Incomplete outcome data addressed	Selective results reporting
Chen 2013	Unclear	Low	Low	Unclear	Unclear	Unclear
Guan 2015	Low	Unclear	Low	Unclear	Unclear	Unclear
Kong 2018	Low	Unclear	Low	Unclear	Unclear	Unclear
Li 2015	Low	High	Low	Low	Low	Low
Mohamed 2014	Unclear	High	High	High	Low	Low
Shi 2020 1	Low	Unclear	Low	Low	Low	Low
Shi 2020 2	Low	Unclear	Low	Low	Low	Low
Wang 2020	Low	Unclear	Low	Low	Low	Low
Xu 2017	High	High	High	High	High	Unclear
Liu 2020	Low	Low	Low	Low	Low	Low
Shan 2015	Low	Unclear	Unclear	Unclear	Low	Low
Ge 2011	Low	Unclear	Unclear	Unclear	Low	Low
Kang 2010	Low	Unclear	Unclear	Unclear	Low	Low
Wang 2017	Low	Unclear	Unclear	Unclear	Low	Low
Xu 2013	Low	Unclear	Unclear	Unclear	Unclear	Unclear
Zhang 2018	Low	Unclear	Low	Low	Low	Low
Pan 2016	Unclear	Unclear	Unclear	Unclear	Low	Unclear
Mu 2017	Low	Low	Low	Low	Low	Low
Liu 2014	Low	Unclear	Unclear	Unclear	Low	Low
Zhang 2019	High	High	High	High	High	Unclear

Continuation table of **Supplementary Table 2**.

	Random sequence generation	Allocation concealment	Blinding of participants and personnel	Blinding of outcome assessment	Incomplete outcome data addressed	Selective results reporting
Tian 2015	Low	Unclear	Unclear	Unclear	Low	Low
Jin 2013	Unclear	Unclear	Unclear	Unclear	Low	Low
Zhang 2014	Unclear	Unclear	Unclear	Unclear	Low	Unclear
Zhao 2012	Low	Unclear	Unclear	Unclear	Low	Low
Zhu 2016	Low	Unclear	Low	Unclear	Low	Low
Wu 2016	Unclear	Unclear	Unclear	Unclear	Low	Low
Mansouri 2019	Unclear	Unclear	Low	Low	Low	Low
Li 2019	Low	Unclear	Low	Low	Low	Low
Lee 2015	Low	High	Low	Unclear	Low	Low
She 2017	Unclear	Unclear	Unclear	Unclear	Low	Low
Zhang 2013	Unclear	Unclear	Unclear	Unclear	Low	Low
Li 2021	Low	Low	Low	Low	Low	Low
Peng 2012	Low	Unclear	Unclear	Low	Unclear	Unclear
Zhang 2014	Low	High	High	High	Low	Low
Cheng 2019	Unclear	Low	Low	High	Low	Low
Zhao 2020	Low	Unclear	Low	Low	Low	Low

Supplementary Table 3. Rank probabilities of each drug in preventing POCD.

Supplementary Table 3A. Rank probabilities of each drug in preventing POCD on postoperative Day 1.

	V1	V2	V3	V4	V5
Placebo	0	0	0.001375	0.082	0.916625
Dexmedetomidine	0.020625	0.17975	0.57475	0.224875	0
Ulinastatin	0.30175	0.48675	0.143375	0.066625	0.0015
Midazolam	0.033625	0.118375	0.20325	0.568125	0.076625
Ketamine	0.644	0.215125	0.07725	0.058375	0.00525

Supplementary Table 3B. Rank probabilities of each drug in preventing POCD on postoperative Day 3.

	V1	V2	V3	V4	V5
Placebo	0	0	0	0.014	0.986
Dexmedetomidine	0.00025	0.070125	0.35725	0.572375	0
Ulinastatin	0.631	0.359625	0.007375	0.002	0
Parecoxib	0.00475	0.145375	0.564625	0.285	0.00025
Ketamine	0.364	0.424875	0.07075	0.126625	0.01375

Supplementary Table 3C. Rank probabilities of each drug in preventing POCD on postoperative Day 7.

	V1	V2	V3	V4	V5	V6
Placebo	0	0	0.0005	0.006375	0.139	0.854125
Dexmedetomidine	0.00575	0.09675	0.512375	0.315	0.06975	0.000375
Ulinastatin	0.3315	0.586375	0.05475	0.021625	0.005625	0.000125
Parecoxib	0.003625	0.054375	0.158	0.288875	0.440375	0.05475
Midazolam	0.013625	0.080375	0.2255	0.316	0.30625	0.05825
Ketamine	0.6455	0.182125	0.048875	0.052125	0.039	0.032375

Supplementary Table 4. Summary odds ratios and heterogeneity for each comparison.

Supplementary Table 4A. Summary odds ratios and heterogeneity for each comparison on postoperative Day 1.

Drug	Comparison	OR (95% CI.)	I^2	P value
	Direct	0.33 (0.23, 0.47)	18.0%	
Dexmedetomidine vs. Placebo	Indirect	NA		-
	Network	0.33 (0.23, 0.46)	18.0%	
	Direct	0.19 (0.052, 0.61)	0.5%	
Ulinastatin vs. Placebo	Indirect	NA		-
	Network	0.19 (0.061, 0.55)	0.6%	
	Direct	0.5 (0.12, 2.0)	-	
Midazolam vs. Placebo	Indirect	NA		-
	Network	0.47 (0.14, 1.4)	-	
	Direct	0.16 (0.014, 2.2)	0.0%	
Ketamine vs. Placebo	Indirect	NA		-
	Network	0.11 (0.017, 0.54)	0.0%	
	Direct	0.63 (0.10, 3.9)	-	
Ulinastatin vs. Dexmedetomidine	Indirect	0.20 (0.0079, 2.0)		0.4148
	Network	0.57 (0.18, 1.7)	0.0%	
	Direct	1.1 (0.21, 6.5)	-	
Midazolam vs. Dexmedetomidine	Indirect	NA		-
	Network	1.5 (0.42, 4.4)	-	
	Direct	0.26 (0.025, 1.5)	-	
Ketamine vs. Dexmedetomidine	Indirect	0.44 (0.043, 4.5)		0.5329
	Network	0.37 (0.039, 1.7)	0.0%	

Supplementary Table 4B. Summary odds ratios and heterogeneity for each comparison on postoperative Day 3.

Drug	Comparison	OR (95% CI.)	I^2	P value
Dexmedetomidine vs. Placebo	Direct	0.39 (0.25, 0.57)	0.0%	-
	Indirect	NA		
	Network	0.40 (0.25, 0.57)	0.0%	
Ulinastatin vs. Placebo	Direct	0.096 (0.033, 0.23)	6.9%	-
	Indirect	NA		
	Network	0.11 (0.042, 0.26)	4.8%	
Parecoxib vs. Placebo	Direct	0.36 (0.20, 0.58)	0.0%	-
	Indirect	NA		
	Network	0.35 (0.20, 0.57)	0.0%	
Ketamine vs. Placebo	Direct	0.17 (0.018, 0.79)	-	-
	Indirect	NA		
	Network	0.16 (0.021, 0.85)	-	
Ulinastatin vs. Dexmedetomidine	Direct	0.70 (0.10, 4.8)	-	0.231
	Indirect	0.18 (0.044, 0.56)		
	Network	0.27 (0.11, 0.73)	0.0%	
Parecoxib vs. Dexmedetomidine	Direct	0.83 (0.21, 3.5)	-	0.8832
	Indirect	0.91 (0.43, 2.1)		
	Network	0.91 (0.49, 1.7)	0.0%	

Supplementary Table 4C. Summary odds ratios and heterogeneity for each comparison on postoperative Day 7.

Drug	Comparison	OR (95% CI.)	I^2	P value
Dexmedetomidine vs. Placebo	Direct	0.43 (0.29, 0.64)	0.0%	-
	Indirect	NA		
	Network	0.42 (0.28, 0.66)	0.0%	
Ulinastatin vs. Placebo	Direct	0.21 (0.093, 0.41)	29.3%	-
	Indirect	NA		
	Network	0.22 (0.089, 0.46)	28.2%	
Parecoxib vs. Placebo	Direct	0.55 (0.30, 1.1)	0.0%	-
	Indirect	NA		
	Network	0.56 (0.30, 1.1)	0.0%	
Midazolam vs. Placebo	Direct	0.33 (0.068, 1.4)	-	0.3973
	Indirect	0.74 (0.22, 2.4)		
	Network	0.50 (0.21, 1.1)	0.0%	
Ketamine vs. Placebo	Direct	38 (0.62, 140)	45.3%	-
	Indirect	NA		
	Network	0.12 (0.0048, 1.1)	59.0%	
Midazolam vs. Dexmedetomidine	Direct	1.3 (0.55, 3.2)	0.0%	-
	Indirect	NA		
	Network	1.2 (0.52, 2.6)	6.5%	
Ketamine vs. Dexmedetomidine	Direct	0.0098 (0.0030, 180)	-	< 0.001
	Indirect	0.31 (0.017, 5.6)		
	Network	0.28 (0.0083, 2.7)	4.6%	

2 Supplementary Figures

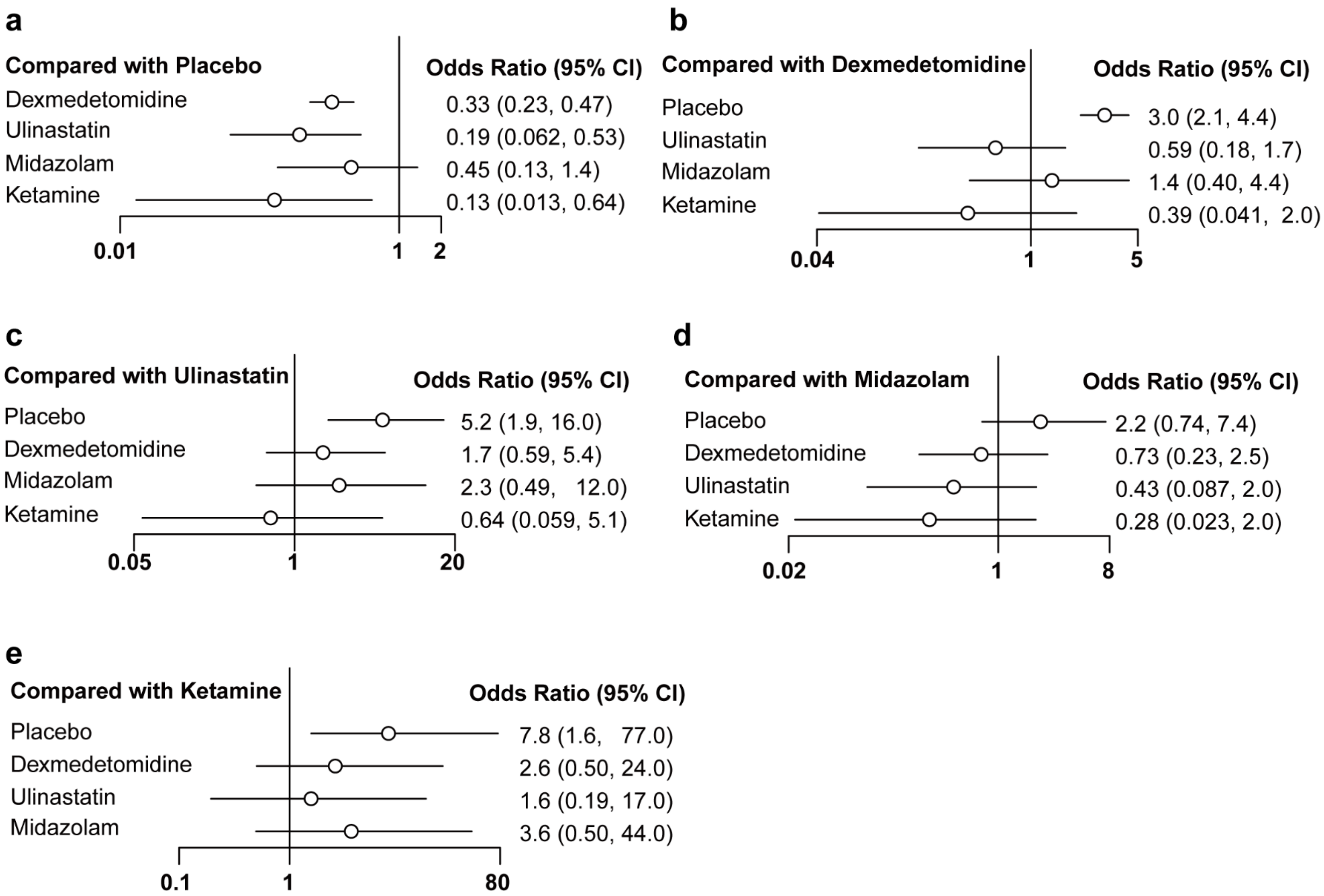

Supplementary Figure 1. Forest plots of odds ratios (95% confidence interval) produced by network meta-analysis postoperative Day 1.

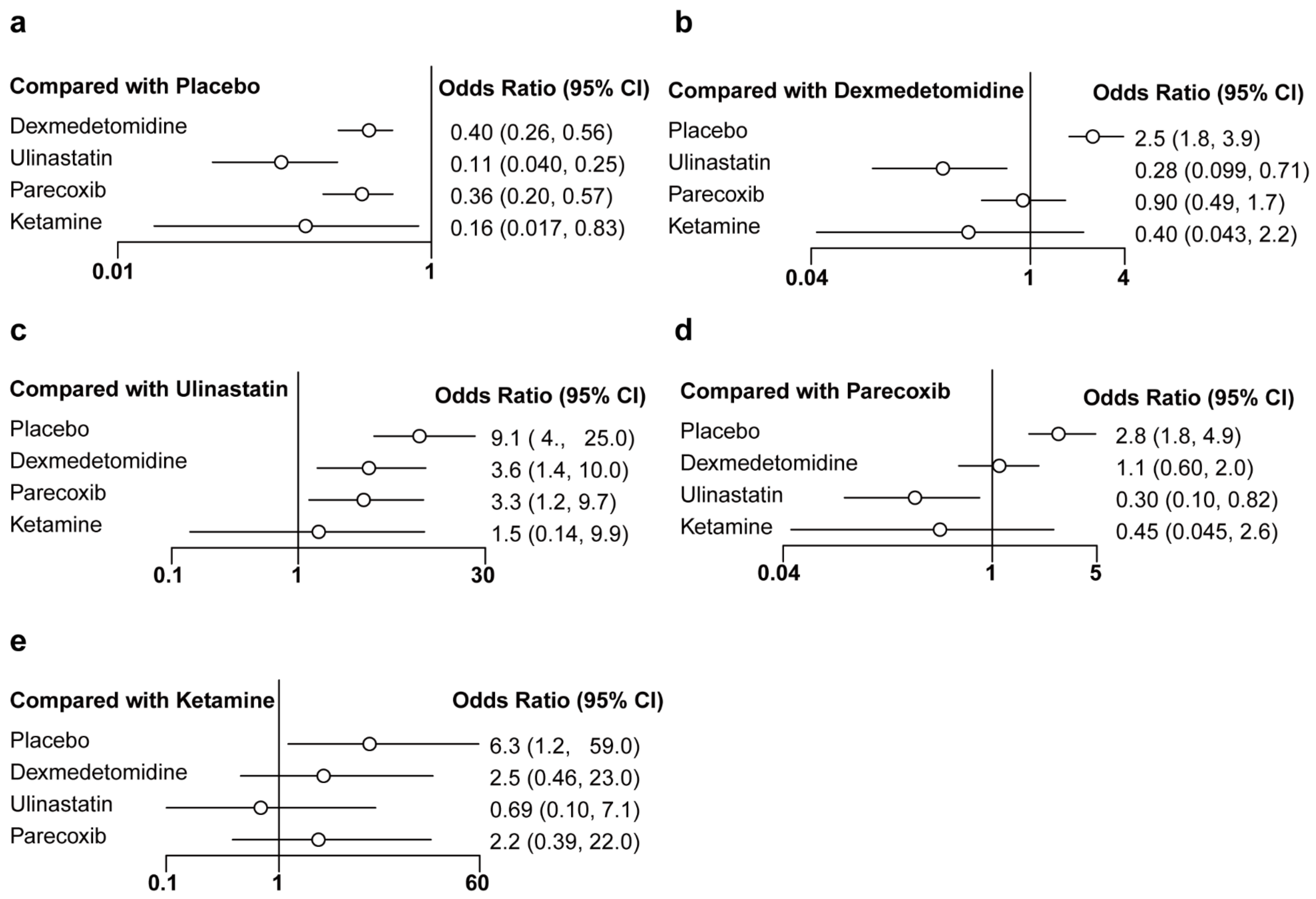

Supplementary Figure 2. Forest plots of odds ratios (95% confidence interval) produced by network meta-analysis postoperative Day 3.

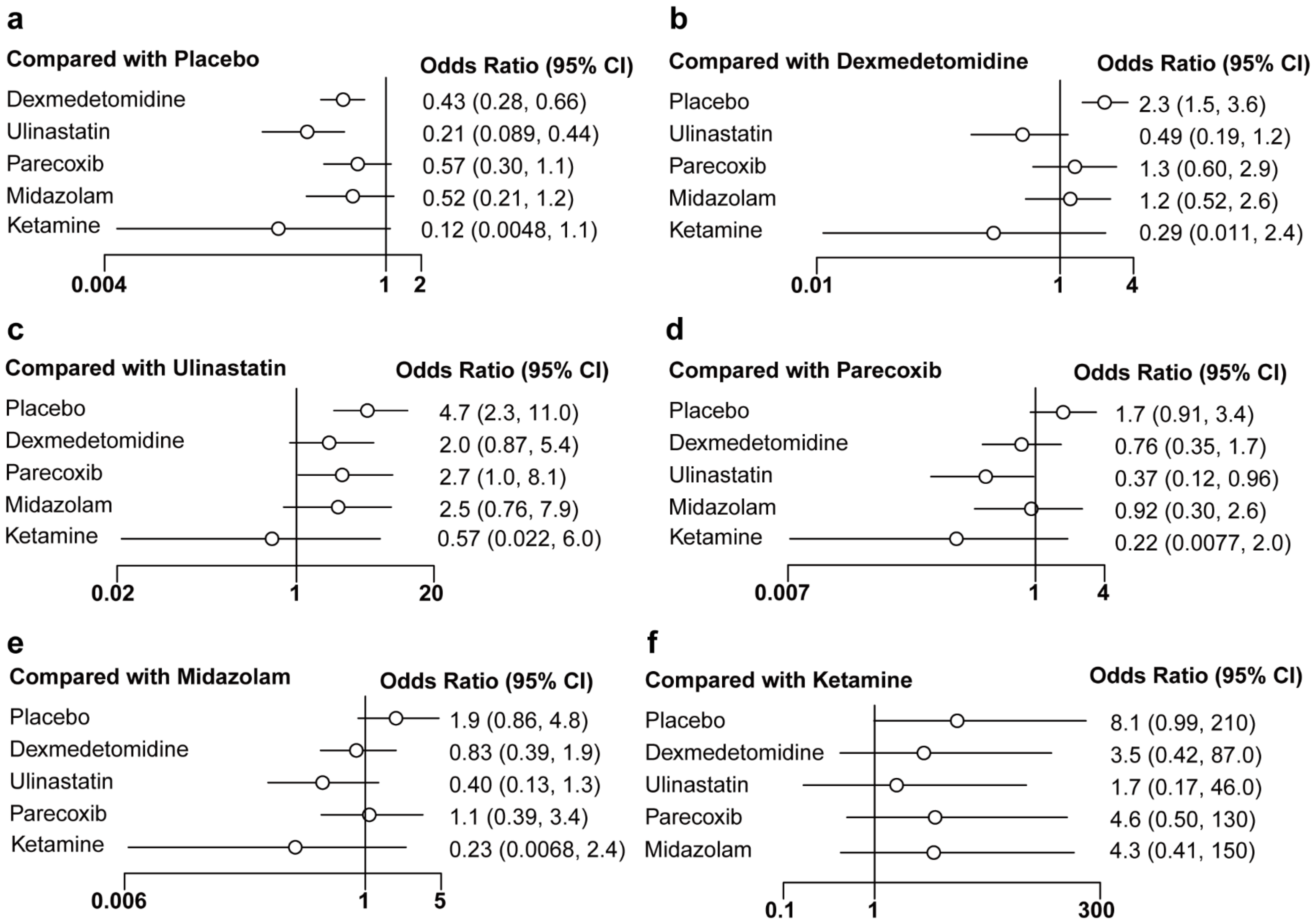

Supplementary Figure 3. Forest plots of odds ratios (95% confidence interval) produced by network meta-analysis postoperative Day 7.
